# The Potential Application of Natural Photosensitizers Used in Antimicrobial Photodynamic Therapy against Oral Infections

**DOI:** 10.3390/ph15060767

**Published:** 2022-06-20

**Authors:** Shima Afrasiabi, Alireza Partoazar, Nasim Chiniforush, Ramin Goudarzi

**Affiliations:** 1 Laser Research Center of Dentistry, Dentistry Research Institute, Tehran University of Medical Sciences, Tehran 1441987566, Iran; shafrasiabi@alumnus.tums.ac.ir; 2 Experimental Medicine Research Center, Tehran University of Medical Sciences, Tehran 1441987566, Iran; partoazar@tums.ac.ir; 3 Division of Research and Development, Pharmin USA, LLC, San Jose, CA 95128, USA

**Keywords:** photosensitizing agents, photochemotherapy, dental infection control, drug resistance, biofilms, nanoparticles

## Abstract

Oral health problems and the emergence of antimicrobial resistance among pathogenic bacterial strains have become major global challenges and are essential elements that negatively affect general well-being. Antimicrobial photodynamic therapy (APDT) is based on a light source and oxygen that activates a nontoxic photosensitizer, resulting in microbial destruction. Synthetic and natural products can be used to help the APDT against oral microorganisms. The undesirable consequences of conventional photosensitizers, including toxicity, and cost encourage researchers to explore new promising photosensitizers based on natural compounds such as curcumin, *chlorella*, chlorophyllin, phycocyanin, 5-aminolevulinic acid, and riboflavin. In this review, we summarize in vitro studies describing the potential use of APDT therapy conjugated with some natural products against selected microorganisms that are considered to be responsible for oral infections.

## 1. Introduction

Oral diseases such as caries, and endodontic–periodontal diseases are prevalent worldwide. The heavy burden of oral infectious diseases on health-related quality of life creates a strong ongoing need for developing more effective therapies with fewer complications [1]. Antibiotics are generally applied in the treatment of oral infectious diseases [1]. Several studies reported irrational antibiotic prescriptions with no scientific reasoning in dental ailments [2,3,4]. There was increasing concern about incorrect and inappropriate use of antibiotics due to bacterial drug resistance [1]. An important risk of the systemic use of antibiotics is superinfection, which makes it impossible to eradicate the pathogen [5]. Systemic use of antibiotics also causes side effects including increased sensitivity, gastrointestinal intolerance, and the development of bacterial resistance, as Rams et al. showed 71.7% resistance to at least one antimicrobial agent in a group of 120 peri-implantitis subjects [6]. Furthermore, systemic use of antibacterial is limited due to the lack of access to periodontal organisms in periodontal pockets [7].

Advanced understandings of the pathological consequences of oral infectious diseases have raised the demand for the design of antibacterial therapeutic strategies [1]. Antimicrobial photodynamic therapy (APDT) was described as a promising antibacterial therapeutic option to overcome the aforementioned drawbacks. APDT is a minimally invasive antimicrobial approach that has been proposed as adjunctive therapy for the treatment of local infections that are resistant to antibiotics [8]. APDT is a procedure that activates photosensitizers in the presence of oxygen to produce free radicals and reactive oxygen species (ROS) that are capable of causing cell death [9,10]. Compared with other conventional antimicrobials, APDT has several strengths, including that APDT is a multitarget process, in which the spread of microbial resistance will be very rare. APDT demonstrated a rapid lethal effect. It is a successful treatment that kills effectively a variety of microorganisms. In addition, it is known as a cost-effective and safe method [11]. Despite the advantages associated with APDT, there are some limitations to using them, including that oral bacteria in biofilms are less affected by APDT than bacteria in the planktonic stage [12]. Furthermore, the lack of selectivity to microorganisms can be also regarded as a limitation of APDT applications [11]. APDT has been reported in dental diseases against periodontal bacteria, and caries-related bacterial strains [13,14,15].

Photosensitizers have a key role in the APDT process as absorbers of light energy [16]. Several factors are taken into consideration when choosing photosensitizers. Photosensitizers should be chemically pure, produced under good production conditions with quality control and low production costs, and lead to better storage stability. These photosensitizers must have a maximum absorption peak in the red spectrum up to the near-infrared range of 650–800 nm because the absorption of single photons with wavelength above 800 nm does not produce enough amount of energy values to stimulate oxygen. Photosensitizers should have significant ease of quantum operation that results in good ROS production after irradiation. They should be non-toxic and rapidly removed from normal tissues to minimize side effects [17].

Natural products are beneficial due to their unique structural, chemical, antimicrobial, and anti-inflammatory properties [18]. In this review, essential functions, and advantages of natural photosensitizer-based APDT against oral infectious are first discussed. Then, a special focus on APDT in combination with nanoparticles (NPs) using natural photosensitizers is presented.

## 2. Mechanism of Action of APDT

**Figure 1:**
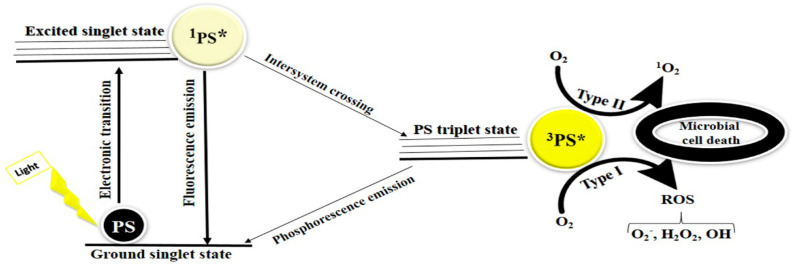
Mechanism of APDT. After absorption of single photon, the photosensitizer transfers from its ground singlet state to an excited singlet state. Next, the photosensitizer can lose energy, thereby returning to ground state, or it can turn to an excited triplet state. The long−lived triplet state can react with oxygen in two ways, as follows: In type I reactions, the charge is transferred to form ROS. In type II reactions, energy is transferred directly to the ground state molecular oxygen (^3^O_2_), leading to the appearance of singlet oxygen (^1^O_2_). ^1^PS*; photosensitizers in its singlet excited state, ^3^PS*; photosensitizers in its triplet excited state.

APDT consists of three main components: a specified wavelength light source, a photosensitizer, and oxygen. In the APDT process, when the photosensitizer components are exposed to a specific wavelength of light, the photosensitizer can lose energy, thereby returning to the ground singlet state, or it can be converted to a long-lived triplet state by intersystem crossing. From this state, it can back to the ground state either via phosphorescence emission, or by two mechanisms generating ROS, as follows: The photosensitizer can interact with oxygen to produce hydrogen peroxide (H_2_O_2_), superoxide anion radical (O_2_), and hydroxyl radicals (^•^OH) (type 1) or interact with oxygen, forming singlet oxygen (^1^O_2_) (type 2) (Figure 1). The products produced in these reactions can cause significant damage to microorganisms and irreversibly alter their metabolic activity, resulting in the death of target cells [16].

## 3. Application of Natural Photosensitizers in Dental Caries

Dental caries is a multifactorial, non-transmissible, and biofilm-mediated disease occurred by phasic de/and remineralization process of susceptible dental hard tissues [19]. The main factors contributing to the pathogenesis are the acidic stage of *Lactobacilli* and *Streptococcus mutans* produce acid through the metabolism of a wide variety of carbohydrates, especially sucrose, and hence causes an acidic pH and enamel demineralization [20]. The treatment of deep carious lesions approximating the vital pulps is challenging. Complete removal of deep caries put the pulp at risk of exposure. Partial removal of dental caries has been advocated for avoiding pulp exposure, as carious dentin is left in proximity to the pulp led to a risk reduction for pulpal exposure [21]. The application of APDT as a complementary step of partial removal of dental caries appears as a favorable therapeutic candidate to stimulate dental practitioners in adopting minimal intervention procedures instead of old-fashioned restorative methods [22]. A photosensitizer penetrates a target cell without causing any adverse effects itself, which is very helpful [23].

Curcumin extracted from *Curcuma longa* is widely used in food and cosmetic industries and as a spice and coloring agent. It has characteristics such as anti-inflammatory, antitumoral, antioxidant, antibacterial, and chemotherapeutic effects, which make it valuable for photobiological application. It can also be used in APDT as a photosensitizer. It has the capacity to produce ROS and free radicals resulting in phototoxicity procedures. Due to its hydrophobic behavior, the same changes in its structure including the production of nanocurcumin make it more applicable than its previous structure. It has a peak absorption in the blue range of electromagnetic wavelengths [24,25,26]. Méndez et al. studied the effects of curcumin on total *Streptococci*, *S. mutans*, and total *Lactobacilli* biofilms, and reported that 600 μmol/L curcumin plus 75 J/cm^2^ light-emitting diodes (LED) reduced significantly the vitality of intact biofilms. In contrast, the results showed that curcumin alone exhibited no change in the vitality of intact biofilms [22]. Likewise, the author reported that curcumin with 2 or 5 min LED irradiation times reduced the vitality of 5-day grown biofilms [27]. Lee et al. suggested that curcumin in combination with *Curcuma xanthorrhiza* (a plant-derived, natural product) can induce a photodynamic reaction under irradiation by 405 nm LED at an energy density of 25.3 J/cm^2^ and effectively inhibit planktonic *S. mutans* cells [28]. Several studies showed no cytotoxicity to fibroblast cells, with curcumin as the photosensitizer [29,30,31].

*Chlorella* is a green natural microalga. It contains proteins, vitamins, and minerals, and is used as a dietary compound. It has antimicrobial, anticancer, anti-inflammatory, and antioxidant wound-healing characteristics. It can be activated by red wavelength [32,33]. Hwang et al. investigated APDT with *Chlorella* or *Curcuma* extracts against *S. mutans* biofilm upon exposure to LED light at 17.7 J (405 nm, 59 mW × 300 s). *Chlorella* and *Curcuma* treatment groups exhibited a 40% and 50% reduction in the live/dead bacteria ratio, respectively, when compared with that in the control group [33].

Chlorophyll, a green pigment that is found in green plants, can be activated by the range of 410–800 nm wavelengths. The high potency of ROS production and structure modification by replanting metal ion complex make it suitable as a photosensitizer in APDT procedures [34,35]. Phycocyanin is a bluish pigment used in the food industry as a coloring agent. This compound is extracted from *spirulina* with antioxidant, immunomodulation, and antimicrobial effects, which makes it a potential photosensitizer in APDT procedures. The peak absorption of this complex is in the red range of wavelengths. When this compound is activated by an appropriate wavelength, it can produce singlet oxygen. Water solubility and non-toxicity nature are two main advantages of this photosensitizer [14,36,37,38]. Our group also reported the APDT effects of 2.4 × 10^−3^ mol/L chlorophyllin–phycocyanin mixture (PhotoActive^+^) with a 3 min exposure to diode laser (635 nm, 104 J/cm^2^) potently reduced the *gtfB* gene expression of *S. mutans*, with rates of 3.5-fold. In addition, PhotoActive^+^–APDT displayed a significant decrease in GtfB protein production of *S. mutans*, by 54% [13]. In addition, according to a previous study, PhotoActive^+^ did not show any cytotoxic effect on human gingival fibroblast [39].

Chlorine e6 (Ce6), which is a derivative of chlorophyll, is mainly used for medical purposes and can be activated by 400–660 nm, but the red one is more acceptable, with an enhanced lifetime in a triplet state and low toxicity. The main mechanism of APDT with this photosensitizer is type II reaction by producing singlet oxygen [40]. Nie et al. showed Ce6 under 200 μM, followed by 660 nm red LED at an energy density of 15 J/cm^2^, resulted in a 5-log reduction in biofilm viability and a 30-fold reduction in biofilm lactic acid production of *S. mutans*. Nevertheless, this study stated that low levels of dark toxicity could be observed when the Ce6 concentration exceeded 50 μM [40].

## 4. Application of Natural Photosensitizers in Endodontic Root Canal Infections

Endodontic diseases are biofilm-associated infections [41]. *Enterococcus faecalis* is one of the most important species isolated from root canals with persistent endodontic infection, which are often difficult to eradicate due to its inherent antimicrobial resistance, biofilm formation, and the ability of dentinal tubule invasion [42]. Microbial biofilms in endodontics are more resistant to disinfecting medicaments used in root canal treatment. The complexity of the root canal system and the multispecies biofilm communities increase the difficulty in an effective eradication of the microbial biofilm [43]. It is believed that if microorganisms are present in the root canal, they may persist, resulting in treatment failure [44]. Hence, endodontic research has focused on developing procedures that can significantly reduce residual bacteria. APDT can be considered a complementary method to act as a support to root canal disinfection [45].

The recent evidence in this regard has been shown by Rocha et al.; after incubation of *E. faecalis* in artificial bone cavities for biofilm formation, APDT was conducted using the photosensitizer curcumin and LED (450 nm, 67 mW/cm^2^, and 20.1 J/cm^2^). Then, microbiological samples from the bone cavities were taken. The results showed curcumin at concentrations of 1.5 g/L significantly reduced the *E. faecalis* growth rate by 1.92 log_10_ colony-forming unit (CFU)/mL in comparison to the control group. In addition, fluorescence spectroscopy images revealed a greater reduction in biofilm in the curcumin–APDT group [46].

In another in vitro study, Diogo et al. examined the antimicrobial activity of the Zn(II) Ce6 methyl ester (Zn(II)e6Me), a chlorophyll-derived photosensitizer against mono and mixed biofilms of *E. faecalis* with *Candida albicans* from infected human dentin discs and root blocks. Bacteria were exposed to red light (627 nm, 75 mW, 3150 J/cm^2^) for 90 s. It was demonstrated that chlorophyll derivative treatment of dentin discs and root block lead to 59.1% and 79.7% biofilm reductions, respectively [47]. A recent investigation revealed that phycocyanin alone and phycocyanin–APDT decreased the viability of *E. faecalis* by 38.1%, and 89.45%, respectively. In addition, phycocyanin-APDT could significantly reduce the *fsrB* expression, by 10.8-fold [48]. Furthermore, an in vitro study found that APDT using 500 μg/mL of *Chlorella* plus 660 nm diode laser at an energy density of 23.43 J/cm^2^ is effective against *E. faecalis* biofilms [49].

## 5. Application of Natural Photosensitizers in Periodontitis

Periodontitis is the most common bacterial infectious disease that has attracted a great deal of public health attention. This disease leads to progressive damage such as gingival atrophy, alveolar bone resorption, and eventually tooth loss, with progressive degeneration of periodontal-supporting tissues [50]. Periodontitis is also associated with the occurrence of many systemic diseases. Therefore, it is necessary to find effective and safe methods in the treatment of this disease [51]. *Porphyromonas gingivalis* and *Aggregatibacter actinomycetemcomitans* are the most common bacteria causing periodontitis. Periodontitis is a potential risk factor for peri-implantitis [52,53]. The effect of many systemic antimicrobials is limited because most antibiotics used in clinical context cannot able to suppress *P. gingivalis* and *A. actinomycetemcomitans* indefinitely. Moreover, systemic treatment may reduce total bacterial count but are unable to eradicate the target organisms located deep in the biofilm [53,54]. In addition, the acceptable efficacy of antimicrobials can only maintain over a short time, and antibiotic-resistant strains can occur during antimicrobial treatment. The bacteria produce a biofilm, a membrane-like structure composed of microbial cells and an extracellular polymer matrix surrounded by exopolysaccharides [54]. The biofilm structure acts as a physical barrier and leads to increased resistance to antimicrobial agents compared with planktonic species [55].

Non-surgical mechanical debridement is still an optional treatment in patients with periodontitis, although in cases where there is severe periodontitis surgical intervention and the use of systemic antibiotics are usually indicated [56]. The use of effective adjunctive therapies such as APDT could be considered to improve the outcomes of non-surgical periodontal treatment [57]. In one study, blue LED irradiation (450–470 nm, output power density 1.2 W/cm^2^) at 6, 12, and 18 J/cm^2^ alone reduced the number of CFU/mL of both *P. gingivalis* and *A. actinomycetemcomitans*, but this reduction did not reach statistical significance. APDT at a concentration level of 20 μmol/L curcumin with blue LED at 18 J/cm^2^ reduced bacterial counts of *P. gingivalis* and *A. actinomycetemcomitans* by 0.43 and 1.51 log_10_ CFU/mL, respectively [58]. Likewise, Zakeri et al. found that curcumin (60 μM, incubation for 5 min) reduced the survival rate of *P. gingivalis* by about 50% [59]. Saitawee et al. reported that APDT with the *Curcuma longa* inhibits the growth of *A. actinomycetemcomitans* when stimulated with 420–480 nm LED at an energy density of 16.8 J/cm^2^ [60].

The in vitro experiments of one study demonstrated that acid-etched (SLA) titanium discs contaminated with *A. actinomycetemcomitans* biofilm treated with phycocyanin mediated APDT is able to significantly reduce *A. actinomycetemcomitans* biofilm by 40.07%. This study compared 635 nm diode laser, and phycocyanin alone, and observed that the efficacy of APDT was superior to both of the mentioned modalities (40.07% in comparison to 15.38 and 27.54%, respectively) [14].

Al-Ahmad et al. investigated APDT using a Guatteria blepharophylla visible light and water infiltrated infrared A (wIRA) in combination with Ce6 against *Eikenella corrodens*, *Actinomyces odontolyticus*, *Fusobacterium nucleatum*, *Parvimonas micra*, *Slackia exigua*, *Atopobium rimae*, *A. actinomycetemcomitans*, and *P. gingivalis* in planktonic phase and within subgingival oral biofilms communities. The authors reported that Ce6 with 5 min of exposure to 200 mW/cm^2^ light + wIRA showed an APDT effect. According to the live/dead staining results, a significant reduction (33.45%) of treated bacterial cells within subgingival biofilm was observed [61].

## 6. Application of Natural Photosensitizers in Orthodontic System

Orthodontic therapy involves the movement of teeth and jaw bones in order to align them and produce harmony among them. The duration of treatment may be long according to the seriousness of the occlusal malalignment. Oral biofilm formation is one of the most common risks of orthodontic treatment due to the difficulty in completely eliminating biofilms through brushing. In addition to a variety of periodontal and surgical applications, APDT can be widely used to treat orthodontic infections that are caused by a profuse bacterial buildup [62].

Riboflavin or vitamin B2 is a yellowish pigment that can be used as a photosensitizer. Due to its capability of singlet oxygen and hydrogen peroxide and derivatives generation upon irradiation by UV or blue wavelengths. It is a non-toxic compound and has a role in providing cellular metabolism [63,64]. In a previous in vitro study, Kamran et al. showed that riboflavin–APDT significantly reduced the amounts of *Streptococcus sanguinis* and *S. mutans* around the orthodontic brackets [62]. Algerban et al. demonstrated that the metabolic activity of *S. mutans* significantly decreased with the addition of a high amount of either rose bengal or riboflavin, with a light source for illumination (375 nm, 3 mW/cm^2^). The metabolic activity of 0.1% rose bengal or riboflavin after APDT reduced to a certain extent on the 30th day, and 0.5% of either rose bengal–APDT or riboflavin–APDT showed relatively reduced viability of *S. mutans* well under 35% [65].

## 7. Application of Natural Photosensitizers in Oral Candidiasis

*C. albicans* is the most commonly encountered oral manifestation, especially in HIV patients [66]. This is due to its adherence abilities and form of biofilms on the oral tissues and denture surfaces [67]. Biofilm-associated *Candida* infections show high resistance to antifungal treatment and host defense mechanisms. Hence, alternative strategies such as APDT against the emergence of drug-resistant *C. albicans* are being considered [66].

Mittal et al. indicated that the use of different laser wavelengths (He–Ne: 633 nm, and Nd-YAG: 532 nm) with output power 17, and 27 mW/cm^2^ in combination with *Beta vulgaris* as a natural photosensitizer are significantly effective on the viability of *C. albicans* [68]. Ma et al. studied the effects of curcumin on *C. albicans* biofilms, performed experimental studies on the standard strain and two clinical isolates from HIV and oral lichen planus, and reported that 6 min of exposure to 7.92 J/cm^2^ LED with the 60 μM curcumin reduced *C. albicans* biofilms. Furthermore, expression of *efg1*, *ume6*, *hgc1*, and *ece1* genes expression of *C. albicans* was decreased after curcumin–APDT [66].

5-aminolevulinic acid (ALA), the natural precursor of protoporphyrin IX (PpIX), presents several advantages, including high matrix penetration, high degree of photostability, lower toxicity, and water solubility, and can be rapidly cleared from the target cells [69,70]. In a study by Shi et al., photoinactivation of 15 mM ALA mediated APDT and inhibited the growth of *C. albicans* biofilms up to 74.45% by increasing the uptake of the protoporphyrin IX in the biofilms. Moreover, ALA–APDT under a 635 nm red light source at an energy density of 300 J/cm^2^ showed potent inhibition of the metabolic activity of *C. albicans* [71]. In addition, the photoactivation of PpIX to combat *Actinomyces* *israelii*, and *F. nucleatum* was investigated, and PpIX had a substantial ability to inactivate these microorganisms [72].

Aloe-emodin is a natural compound isolated from *Aloe vera* and *Rheum palmatum*. Ma et al. evaluated the potential application of the aloe-emodin for drug-resistant *C. albicans* strains and found aloe-emodin in a concentration of 10 μM plus 400–780 nm LED at an energy density of 4.8 J/cm^2^ to be an effective photosensitizer in APDT [73].

## 8. Application of Natural Photosensitizers in NP-Based APDT

NPs are known as the main products of nanotechnologies, with diameters of 100 nm or less [74]. NPs possess unique properties, compared with their bulk material, that can overcome the drawbacks of photosensitizers, including low water solubility, uncontrollable photosensitizer release, poor target selectivity, and low extinction coefficient, which has limited their clinical application [1].

Sun et al. investigated the antimicrobial and antibiofilm activities of NPs containing Ce6, coumarin 6 (C6), and Fe_3_O_4_ NPs via APDT against a panel of periodontitis pathogens including *S. sanguinis*, *P. gingivalis*, and *F. nucleatum*. The Fe_3_O_4_ silane@Ce6/C6-mediated APDT had a much greater reduction in biofilms than the untreated bacteria. For each species, Fe_3_O_4_-silane@Ce6/C6 NPs without light irradiation had similar metabolic activity to the untreated bacteria. The authors showed that Fe_3_O_4_-silane@Ce6/C6 NPs with red-light irradiation (630 nm) exhibited significantly lower metabolic activity than NPs without irradiation. In fact, ^1^O_2_ generated by Fe_3_O_4_- silane@Ce6/C6 NPs with 630 nm light could penetrate into dental plaque and play a great role in the antimicrobial effect against periodontal pathogens. In addition, the L929 mouse fibroblast cells were more than 80% viable with a concentration of 10 μM of Fe_3_O_4_- silane@Ce6/C6 NPs [75].

Graphene quantum dots (GQDs), which are carbon-based of nanometer size, owing to their excellent water solubility, photostability, non-toxicity, biocompatibility, etc., thus making them beneficial for a wide variety of applications in nanomedicine [76]. Mushtaq et al. reported that curcumin loading on GQDs is expected to resolve the poor water solubility issue of curcumin but also enhance ROS production, as the photosensitizer activity of curcumin and GQDs will be combined. Therefore, the loading of curcumin on GQDs can potentially enhance its antimicrobial effects when irradiated with light of a specific wavelength. The results of this study showed that, at a light exposure of 30 J/cm^2^, curcumin-GQDs mediated APDT caused 3.82 log_10_ CFU/mL reduction in *C. albicans*, while GQD–APDT and curcumin–APDT resulted in 1.07 and 1.34 log_10_ CFU/mL reductions in *C. albicans*, respectively. The improved antimicrobial activity of curcumin-GQDs is believed to contribute to the generation of ROS, causing damage to bacterial cell death. Nanoscale photosensitizer delivery system can modify the poor solubility of curcumin in addition to increasing targeted delivery of curcumin and, subsequently, resulting in increased bioavailability of curcumin [77].

Propolis is also a natural product that has gained attention in the scientific community due to its antibacterial, antiviral, and anti-inflammatory activities [78]. In a previous study, the authors used propolis NP (PNP) to investigate the individual and synergistic effects of PhotoActive^+^ as a natural photosensitizer in combination with PNP plus diode laser with the energy density of 103.12 J/cm^2^ in the APDT process against *S. mutans*. The PhotoActive^+^–PNP–APDT significantly suppressed the *S. mutans* biofilm formation by 58%. The expression of *gtfB*, *gtfC*, and *ftf* genes showed a significant reduction after APDT in the presence of PhotoActive^+^ and PNP of about twofold; compared with PhotoActive^+^–APDT, this difference was significant. It could be concluded that the PNP could significantly enhance the APDT outcomes against *S. mutans.* These findings might provide an opportunity for the efficient treatment of localized microbial infections [39]. Zhang et al. combined Ce6 with upconversion NPs (UCNPs) NaYF4: Yb, Er via the amphiphilic silane method. Enhanced bacteriological outcomes were found on *Prevotella. intermedia*, *F. nucleatum*, and *P. gingivalis* and the corresponding biofilms after 980 nm near-infrared light irradiation. The energy transfer triggering due to the existence of Ce6 in the UCNPs is very important for high-efficient APDT [79]. Another example of new drug delivery systems is the use of liposome-based carriers due to the encapsulation ability of both hydrophilic and lipophilic drugs and enhancing the drug action in vivo [80]. Yang et al. suggested cationic liposomes embedded with cetyltrimethylammonium bromide (CTAB) with strong antimicrobial activity, in which Ce6 was encapsulated as a photosensitizer to administer APDT against *C. albicans*. Ce6-loaded CTAB–liposomal compounds showed greater APDT efficacy against *C. albicans*. Encapsulation of photosensitizers in liposomes can retain the photosensitizer in its monomeric form, thereby ensuring high yields of ^1^O_2_ under light irradiation [81].

## 9. Advantages and Limitations

A variety of conventional photosensitizers with lasers of various wavelengths have been used in clinical trials, including toluidine blue, indocyanine green, and methylene blue. Although these photosensitizers have shown promising results in clinical studies, there are limitations to using them [82,83,84]. Natural photosensitizers have been applied directly in APDT without the different synthetic strategies to produce synthetic photosensitizers, which enables the development of new APDT techniques at a lower cost. Many natural photosensitizers are obtained from edible plants and do not require hazardous materials for building up synthetic photosensitizers. Natural photosensitizers are environmentally friendly. The low solubility of natural photosensitizers, low triplet quantum yield upon irradiation, and poor absorption, distribution, metabolism, and excretion properties are major problems limiting the widespread use of natural photosensitizers in APDT [16]. The poor solubility of photosensitizers causes bioavailability problems, susceptibility to hydrolytic degradation, and aggregation before interaction with the target site. This aggregation causes fluorescence quenching and low generation of ROS, resulting in a low APDT efficiency [85]. However, there are not many studies on the use of natural photosensitizers in clinical practice, and studies in this field should be continued.

## 10. Conclusions and Perspectives

In this paper, we focused on the preliminary in vitro studies concerning the efficiency of antimicrobial photoinactivation based on natural photosensitizers against some microorganisms responsible for oral diseases. This review stated several natural products that were found to have demonstrated effective antibacterial photodynamic actions. The known structures of natural photosensitizer molecules open up wide opportunities for their directed modification in order to obtain highly stable derivatives with enhanced photophysical features and increased hydrophilicity, as well as to create conjugates with other molecules that need to be addressed in future studies. These preliminary studies motivate researchers to continue the research of infection control with natural photosensitizers activated by APDT.

## Data Availability

No new data were created or analyzed in this study. Data sharing is not applicable to this article.
